# The IgM pentamer is an asymmetric pentagon with an open groove that binds the AIM protein

**DOI:** 10.1126/sciadv.aau1199

**Published:** 2018-10-10

**Authors:** Emiri Hiramoto, Akihisa Tsutsumi, Risa Suzuki, Shigeru Matsuoka, Satoko Arai, Masahide Kikkawa, Toru Miyazaki

**Affiliations:** 1 Laboratory of Molecular Biomedicine for Pathogenesis, Center for Disease Biology and Integrative Medicine, Faculty of Medicine, The University of Tokyo, Tokyo 113-0033, Japan.; 2 Department of Cell Biology and Anatomy, Graduate School of Medicine, The University of Tokyo, Tokyo 113-0033, Japan.; 3 AMED-CREST, Japan Agency for Medical Research and Development, Tokyo, Japan.; 4 Max Planck–The University of Tokyo Center for Integrative Inflammology, Tokyo, Japan.

## Abstract

Soluble immunoglobulin M (IgM) forms a pentamer containing a joining (J) chain polypeptide. While IgM pentamer has various immune functions, it also behaves as a carrier of circulating apoptosis inhibitor of macrophage (AIM; also called CD5L) protein that facilitates repair during different diseases. AIM binds to the IgM pentamer solely in the presence of the J chain. Here, using a single-particle negative-stain electron microscopy, we found that the IgM pentamer exhibits an asymmetric pentagon containing one large gap, which is markedly different from the textbook symmetric pentagon model. A single AIM molecule specifically fits into the gap, cross-bridging two IgM-Fc that form the edges of the gap through a disulfide bond at one side and a charge-based interaction at the other side. The discovery of the bona fide shape of the IgM pentamer advances our structural understanding of the pentameric IgM and its binding mode with AIM.

## Teaser

We demonstrate the bona fide 2D structure of the IgM pentamer and its association with AIM protein.

## INTRODUCTION

Natural immunoglobulin M (IgM) forms a predominantly pentameric complex that also contains a small polypeptide joining (J) chain (*1*–*5*). The IgM pentamer exhibits a variety of immune responses that are both beneficial and detrimental. For instance, IgM plays a crucial role in the initial defense against foreign pathogens (*6*, *7*) and modified self-components such as cancer cells (*8*–*12*) but is also implicated in the central pathogenesis of some autoimmune diseases (*13*–*15*). Monomeric IgM consists of 14 immunoglobulin domains (two sets of V_H_-Cμ1-Cμ2-Cμ3-Cμ4/V_L_-C_L_) in four polypeptide chains that form a pentameric component (fig. S1). The pentamer also contains an additional polypeptide, the J chain, which assembles the pentamer by firmly bridging the cysteine residues within the C-terminal region of two neighboring IgM monomers (fig. S1) (*2*–*5*, *16*, *17*). The widely accepted conventional structural model of pentameric IgM is primarily based on negative-stain electron microscopy (EM) images reported by Feinstein and Munn (*18*) and later by Shulman and colleagues (*19*), which suggested a star-shaped, symmetric pentagonal structure (fig. S1). More recently, Czajkowsky and Shao (*20*) proposed an improved three-dimensional (3D) model based on analysis from cryo–atomic force microscopy, demonstrating that the IgM pentamer is a nonplanar, mushroom-shaped molecule with a flexural bias. They, as well as some other studies regarding the IgM structure (*21*–*23*), also invoked the symmetric pentagon model as the basic pentameric IgM structure. Unexpectedly, however, our current observation using a single-particle EM of the negatively stained IgM pentamer and the analysis of reference-free 2D class averages of the observed structures indicate that the symmetric pentagon model must be revised.

## RESULTS

### The 2D structure of IgM pentamer

**Fig. 1:**
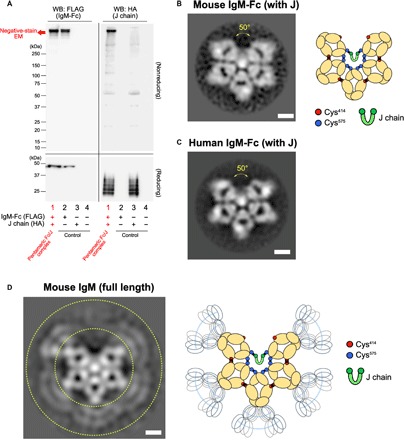
The 2D structure of IgM pentamer. (**A**) Western blotting (WB). FLAG-tagged mouse IgM-Fc and HA-tagged J chain were expressed in HEK293T cells, and the supernatant was analyzed for IgM-Fc (using an anti-FLAG antibody) or the J chain (using an anti-HA antibody) in reducing and nonreducing conditions. As controls, supernatants from cells expressing either or neither molecule were also blotted. Note that the J chain exhibited several bands in a reducing condition with unknown reason. Figure S2 shows the whole picture of the Western blotting in a reducing condition. (**B**) The negative-stain EM image for the mouse IgM-Fc (plus J chain) and a scheme for the image. Scale bar, 5 nm. Each Ig-like polypeptide (Cμ domain; yellow), J chain (green), and the cysteine residues involved in pentameric formation of Fc monomers (red, Cys^414^; blue, Cys^575^) as well as those in the J chain (green) are presented. Black line depicts the disulfide bond. Arrow indicates the specific gap. The central white spot is likely to be an assembly of the tail peptide of each Fc chain (19 amino acids by 10 pieces). (**C**) The negative-stain EM image for human IgM-Fc pentamer. Scale bar, 5 nm. (**D**) The negative-stain EM image of monoclonal mouse IgM pentamer and a scheme for the image. Scale bar, 5 nm. The peripheral region corresponds to Fab (gated by dotted lines), which appears to move flexibly and structurally unlocked and thus could not be observed clearly. In (B) to (D), all particles were picked up in a reference free fashion using Gautomatch.

**Fig. 2:**
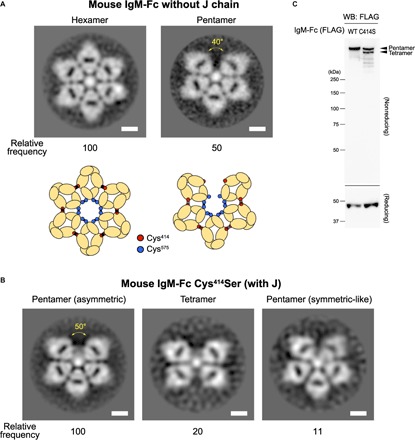
The structure of oligomeric IgM in the absence of J chain. (**A**) The negative-stain EM images for the hexamer and pentamer composed of FLAG-tagged mouse IgM-Fc monomers in the absence of the J chain and their schemes. The frequency of hexamer and pentamer observed in the analysis is described (relative to hexamer as 100). Scale bars, 5 nm. In the scheme, unlike in the presence of the J chain, the disulfide bonds between Cys^414^ residues may not be partly developed (*21*, *25*), and they are drawn by shadowed lines. (**B**) The negative-stain EM images for the pentamer composed by the FLAG-tagged mouse IgM-Fc Cys^414^Ser variant in the presence of the J chain, which include asymmetric pentamers, tetramers, and symmetric-like pentamers. Scale bars, 5 nm. The frequency of observed each item is described (relative to asymmetric pentamer as 100). The size of the gap in the asymmetric pentamer was comparable to that in the wild-type IgM-Fc pentamer. In (A) and (B), all particles were picked up in a reference-free fashion using Gautomatch. (**C**) Western blotting (WB). FLAG-tagged wild type (WT) of Cys^414^Ser (C414S) mouse IgM-Fc and HA-tagged J chain were expressed in HEK293T cells, and the supernatant was analyzed for by an anti-FLAG antibody in nonreducing (top) and reducing (bottom) conditions. Cys^414^Ser IgM-Fc formed both pentamer and tetramer as judged by size as indicated.

Since portions of the Fc region (Cμ2-Cμ3-Cμ4 chains and the short tail region) and J chain are sufficient to assemble monomers and form pentamers correctly (*24*, *25*), we first analyzed a recombinant mouse IgM-Fc pentamer consisting an N-terminal, FLAG-tagged recombinant mouse Fc (Cμ2 to tail) and a hemagglutinin (HA)–tagged J chain, which was produced by cotransfecting both expression plasmids into human embryonic kidney (HEK) 293T cells. Western blotting of the supernatant from transfected cells confirmed the presence of a pentamer containing the J chain (Fig. 1A; the whole picture of the blot in a reducing condition is presented in Fig. 2). The recombinant pentamer purified by an anti-FLAG antibody affinity column and gel fractionation was visualized by negative-stain EM using the RELION analysis software. Unexpectedly, we observed a pentamer-shaped asymmetric pentagon with a large gap, like a hexagon with one piece missing (Fig. 1B, left). The angle of the gap was approximately 50°. Figure S3 shows the nonprocessed images (fig. S3), as well as the results of first and second classification (fig. S4A). We obtained similar results when analyzed using RELION, cisTEM, or Xmipp software (fig. S5). The shapes classified at higher averages were essentially identical, suggesting that the asymmetric pentagon structure was stably formed. The central white spot is likely to be an assembly of the tail peptide of each Fc chain (19 amino acids by 10 pieces). For simplicity, Fig. 1B (right) depicts a scheme for the new pentameric structure based on both the current finding and previous evidence. We also examined recombinant human IgM-Fc pentamer. The human Fc pentamer exhibited an asymmetric pentagon with a 50° gap, identical to the mouse Fc pentamer (Fig. 1C and figs. S3 and S6). When the native IgM pentamer, a hybridoma-derived mouse monoclonal IgM that had an endogenous J chain, was imaged, we observed an asymmetric pentagon similar to the recombinant Fc pentamer (Fig. 1D and figs. S3 and S7A). The Fab domain, which is known to be located at the extremity of each Fc arm, was not clearly observed by negative-stain EM, suggesting that the Fab portion might move flexibly and be structurally unlocked. We obtained similar results when analyzed using RELION (fig. S7A) or cisTEM (fig. S7B).

### Involvement of the J chain and the Fc Cys^414^ residue in the normal pentamer formation

It is likely that the J chain associates with the two monomers at both edges of the gap through cysteines at the Fc tail (Cys^575^), although this cysteine residue makes disulfide bonds to link monomers in other sites (Fig. 1B, right) (*19*, *26*–*29*). This J binding might disturb hexagon formation with an additional IgM monomer, which requires open Cys^575^ residues. In support of this idea, we primarily classified J-negative Fc oligomers visualized by negative-stain EM as symmetric hexagons (Fig. 2A and figs. S3 and S8). Under negative-stain EM imaging, we also observed a pentagon (Fig. 2A and fig. S8), which was predicted by past biochemical experiments, showing that monomeric IgM complexes exist as both hexamers and pentamers in the absence of J chains (*2*, *5*, *30*–*32*). Although the pentagon was asymmetric similar to the J-positive pentamer, the gap was obviously narrower (approximately 40°) than that of the J-positive pentamer. Thus, the five monomers were more closely packed in the presence of a J chain than in the absence of a J chain. This effect might enable five Fc monomers to create additional disulfide bonds with their neighbors using the cysteine resides within the Cμ3 chain (Cys^414^), providing a mechanism for stable assembly (Fig. 1B, right) (*24*, *28*). Consistent with this hypothesis, EM analysis revealed that a variant IgM-Fc in which Cys^414^ was replaced by serine (Cys^414^Ser) could not form the oligomers stably even in the presence of the J chain, forming structures that include tetramers, symmetric pentamers, and asymmetric pentamers with a gap comparable to that in wild-type Fc pentamer (Fig. 2B and figs. S3 and S9). The Western blotting demonstrates that tetramer and pentamer were formed in the absence of disulfide bonds between Cys^414^ residues (Fig. 2C), which is consistent with previous biochemical studies (*24*).

### One apoptosis inhibitor of macrophage molecule fits into the 50° gap

Apoptosis inhibitor of macrophage (AIM; encoded by *cd5l* gene) is a circulating protein belonging to the scavenger receptor cysteine-rich (SRCR) superfamily (*33*), members of which share a highly conserved cysteine-rich domain of approximately 100 amino acids (the SRCR domain) (*34*). Although AIM was initially identified as a supporter of macrophage survival (*33*), accumulating evidence has demonstrated that AIM plays a role in the prevention of a broad range of diseases, such as obesity (*35*, *36*), fatty liver disease (*37*), hepatocellular carcinoma (*37*, *38*), multiple sclerosis (*39*), fungus-induced peritonitis (*40*), and more recently, acute kidney injury (*41*). In the serum, AIM associates with IgM pentamers in the Fc region, which protects AIM from renal excretion and maintains high levels of circulating AIM (approximately 5 μg/ml in humans and mice) (*42*, *43*). However, the binding mode of AIM and the IgM-Fc pentamer remains unclear. Although IgM-bound AIM is functionally inactive, AIM dissociates from IgM during different diseases locally or systemically to exert a function that facilitates disease repair (*43*, *44*). Thus, IgM controls the number and activity of circulating AIM molecules to defend against variable diseases (*41*, *43*).

**Fig. 3:**
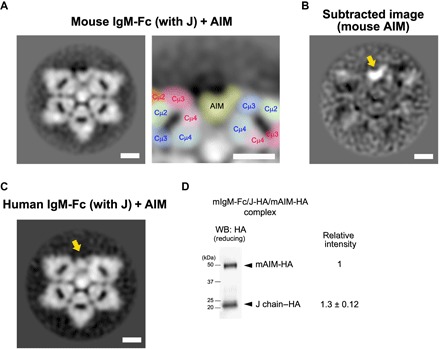
The defined mode of AIM association with pentameric IgM. (**A**) The negative-stain EM image for the mouse IgM-Fc pentamer (with the J chain) associated with AIM. Scale bars, 5 nm. The gap area of the Fc-pentamer carrying a broad bean–like structure (that represents the associated AIM) is also presented at a higher magnification with identification of different Cμ domains by colors. All particles were picked up in a reference-free fashion using Gautomatch. (**B**) An image subtraction between Fc pentamer with and without AIM association. The associating AIM is a broad bean–like structure. Scale bar, 5 nm. (**C**) The negative-stain EM image for the FLAG-tagged human IgM-Fc pentamer with the J chain, associated with the human AIM. Scale bar, 5 nm. All particles were picked up in a reference-free fashion using Gautomatch. (**D**) The number of AIM molecule associated with an Fc pentamer. The Fc-pentamer/J-HA/AIM-HA complex protein was analyzed by Western blotting using an anti-HA antibody in a reducing condition. The AIM-HA band was observed at around 50 kDa in size, whereas the J chain was at around 20 kDa in size. Although the J chain exhibited several bands in a reducing condition, as also described in the legend for Fig. 1A, the number of the band decreased after the column purification. The signal intensity of the bands for AIM-HA and J-HA was quantified using the National Institutes of Health (NIH) ImageJ software. We performed five experiments. The representative blot and the average of the ratio of signal levels are presented.

We therefore examined how AIM associates with the IgM pentamer by visualizing the AIM/IgM-Fc complex. As shown in Fig. 3A, we observed a broad bean–shaped structural associating with an Fc-Cμ4 domain at one edge of the 50° gap and with an Fc-Cμ3 domain at the opposite edge, which was not present in the Fc pentamer in the absence of AIM. An image subtraction of the AIM-negative Fc pentamer from the AIM-positive Fc pentamer supported this observation (Fig. 3B). Human AIM also associated with the human Fc pentamer in a similar fashion (Fig. 3C). We attempted to biochemically quantify the number of AIM molecule associated with each pentamer. We purified the recombinant AIM-bound Fc pentamer in which both AIM and the J chain were tagged with a HA-tag using an anti-FLAG affinity gel column and an anti-AIM antibody column. We immunoblotted the resulting IgM-Fc/J-HA/AIM-HA complex protein using an anti-HA antibody to evaluate the number of AIM molecules associated with one pentamer by quantitating the HA signal of AIM-HA relative to that of J-HA, as one pentamer binds a single J chain (*45*–*47*). The level of HA signal from AIM-HA was almost equivalent to or slightly less than that of J-HA (AIM versus J, 1:1.3 ± 0.12), which denied the presence of more than one AIM molecule within the gap. Thus, it is most likely that a single AIM molecule associated with each pentamer (Fig. 3D).

### The binding mode of AIM to the 50° gap of IgM pentamer

**Fig. 4:**
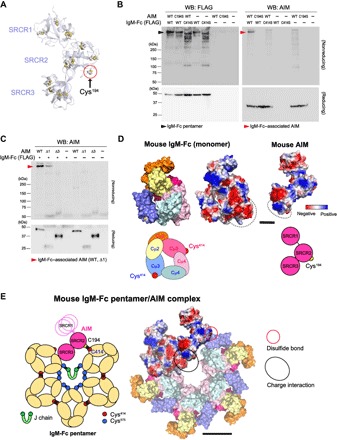
Involvement of a disulfide bond and the charge distribution in AIM-IgM association. (**A**) A homology-based structural model of AIM. Yellow circles indicate cysteine residues. All cysteines except the solitary cysteine at the SRCR2 domain (Cys^194^) form internal disulfide bonds within each SRCR domain. The structural model of AIM and IgM-Fc were built on the basis of Protein Data Bank codes 5a2e and 1o0V. (**B**) Western blotting of the supernatant from cocultured HEK293 cells expressing FLAG-tagged wild-type (WT) or Cys^414^Ser (C414S) variant mouse Fc (with the Myc-tagged J chain) and those expressing wild-type or Cys^194^Ser (C194S) variant AIM as indicated combinations, for Fc (using an anti-FLAG antibody) and AIM (using an anti-AIM antibody), in a nonreducing and reducing conditions. The Cys^414^Ser Fc variant formed various sizes of oligomers (lanes 3 and 5 in the nonreduced IgM blotting). The loading amount per lane was identical (10 μl of supernatant). (**C**) The supernatant from cocultured HEK293 cells expressing FLAG-tagged wild-type (with the Myc-tagged J chain) and those expressing AIM of wild-type, AIM-deleted SRCR1 (Δ1) or AIM-deleted SRCR3 (Δ3), was immunoblotted for AIM using an anti-AIM antibody in a nonreducing and reducing conditions. Note that the expression of Δ1 in the supernatant was lower than others, as observed in the reduced blots. Nevertheless, the binding to Fc pentamer was obvious in contrast to Δ3 that exhibited no binding to IgM-Fc pentamer. (**D**) 3D mapping of the charge distribution of amino acids for mouse IgM-Fc monomer and mouse AIM on the homology-based models. Scale bar, 2 nm. For IgM-Fc, the homology-based model in which each Cμ domain is identified by different color is also shown for obvious orientation. The simplified schemes for IgM-Fc and AIM are also presented. Blue, positively charged; red, negatively charged; white, neutrally charged. The cluster of negatively charged amino acids in IgM Cμ4 domain(s) and positively charged amino acids in AIM SRCR3 domain (including His^294^, Lys^298^, Arg^300^, Lys^301^, and Lys^340^) are gated. Note that the tail chain of the Fc is not included in the model. (**E**) Hypothetic schema of how AIM associates with the IgM-Fc pentamer. Left: A simplified scheme. The AIM containing three SRCR domains is depicted schematically (pink). Yellow circle indicates the solitary cysteine residue at the SRCR2 domain (Cys^194^) of AIM. The SRCR1 domain may move flexibly. Right: A model depicted by reflecting the information of homology modeling and the charge distribution. The charge distribution is reflected only in the AIM molecule and the associating Fc-monomer, whereas other Fc units are colored for different Cμ domains as in (D). Red circle indicates the disulfide bond between the Cys^194^ of AIM and the Cys^414^ of the Fc Cμ3, and black circle indicates the charge interaction between AIM-SRCR3 and the Fc Cμ4. Note that the tail chain of the Fc and the J chain are not included in the model. Scale bar, 5 nm. The size and the structure of the homology model matches the averaged EM images presented in Figs. 1 and 3. In (D) and (E), all molecular graphics representations were created using PyMOL Molecular Graphics System (www.pymol.org).

We then assessed how AIM fits into the 50° gap by performing various biochemical experiments. Since AIM did not dissociate from the IgM pentamer during SDS–polyacrylamide gel electrophoresis in a nonreducing condition, it is likely that a disulfide bond has a function in binding. AIM consists of three SRCR domains (*33*). In mouse AIM, a solitary cysteine residue is found in the SRCR2 domain (Cys^194^), and other cysteine residues form internal disulfide bonds according to a homology-based structural model of AIM using the crystallographic structure of a conserved SRCR domain present in CD6 (Fig. 4A) (*48*). On the other hand, two Cys^414^ residues are free at the edge of the 50° gap in the IgM-Fc pentamer (Fig. 1B, simplified scheme). It is possible that one free Cys^414^ is occupied by the J chain in some circumstances, as Shulman and colleagues (*27*) suggested, leaving one Cys^414^ residue free. Thus, the AIM-IgM association most likely occurs via a disulfide bond between Cys^414^ in the Fc-Cμ3 domain and the Cys^194^ in the SRCR2 domain of AIM. Therefore, the portion of the structure observed to be associating with an Fc-Cμ3 domain at the edge of the 50° gap (Fig. 3A) is likely to be AIM-SRCR2. Supporting this hypothesis, both variant AIM in which the Cys^194^ at the SRCR2 was replaced with a serine (AIM-Cys^194^Ser) and the Cys^414^Ser Fc oligomers were unable to form the AIM/IgM complex (Fig. 4B). The observation that the asymmetric pentamer derived from Cys^414^Ser Fc variant, which contains a 50° gap similar to the wild-type Fc pentamer (Fig. 2B), did not associate with AIM also supports the requirement of Cys^414^ for this association.

In addition, AIM lacking SRCR1 associated with the Fc pentamer (Fig. 4C). This finding is in contrast to AIM lacking SRCR3, which cannot stably bind to the pentamer (Fig. 4C) (*49*). Note that expression level of the AIM lacking SRCR1 was lower than wild-type AIM, suggesting that the SRCR1 domain might contribute to the protein production efficiency and/or the protein stability but not to the binding to IgM. Along with the EM image for the AIM/IgM complex (Fig. 3A), the SRCR3 domain likely interacts with the Fc-Cμ4 domain. This interaction is not mediated by disulfide bonds, as no free cysteines are present in the SRCR3 domain of the mouse AIM or Fc-Cμ4 domain. Thus, it is possible that the interaction is supported by the charge distribution of amino acids, as the SRCR3 domain harbors a surface cluster of positively charged amino acids (including His^294^, Lys^298^, Arg^300^, Lys^301^, and Lys^340^), while the Fc-Cμ4 domain is negatively charged (Fig. 4D). The hypothesis is consistent with our recent finding that feline AIM exhibits a far higher binding affinity for IgM than does human or mouse AIM due to the larger number of positively charged amino acids within the SRCR3 domain (*50*). The SRCR1 domain did not interact with Fc and thus may move flexibly. For this reason, the SRCR1 domain might be unclear by negative-stain EM, similar to the Fab portion of IgM (Fig. 1D). This may be supported by the long hinge region between the SRCR1 and SRCR2 domains compared to that between the SRCR2 and SRCR3 domains (*33*). Together, Fig. 4E shows the overall scheme of how AIM may fit to the gap: A simplified scheme (left) and one depicted reflecting the information of the homology-based structural models (*48*, *51*) and the charge distribution (right) are demonstrated.

## DISCUSSION

In this study, we presented the bona fide shape of pentameric IgM, which is an asymmetric pentagon with a 50° gap. We summarized our major findings in fig. S10. We certainly benefited from using single-particle negative-stain EM and reference-free 2D class average analysis, which provided many advantages in terms of analyzing the pentameric structure more precisely and easily than in previous studies. In the 2D images, an asymmetric pentagon appears less structurally stable than a symmetric pentagon or hexagon. The hexagon formed in the absence of the J chain even exhibits increased hemolytic activity compared to the J-positive pentamer (*52*). Hence, one might wonder why IgM forms an asymmetric pentagon. There are two plausible reasons that explain the formation of an asymmetric pentagon structure: the presence of the J chain, which appears to disturb the hexagon formation, and/or the development of disulfide bonds at the Cys^414^ in the Fc-Cμ3 domain between neighboring monomers. The J chain may closely compact the five monomers to facilitate the disulfide bond at all Cys^414^ residues. Thus, this irregular, asymmetric structure may contribute to the formation of a concrete assembly of five Fc monomers to achieve structural stability. The hexamer may contain a more strained and unnatural 3D structure than the J-positive pentamer, which might cause a functional disadvantage in the alignment of multiple Fab portions in the same orientation toward an antigen. This may decrease the overall affinity of the hexamer for the antigen. Comparative 3D analysis of the IgM-Fc and specific functional assays in the presence or absence of the J chain is needed to clarify this issue. It is also necessary to confirm three dimensionally whether such asymmetric IgM pentamer exhibits the mushroom-shaped structure, which was reported by Czajkowsky and Shao (*20*).

While a more precise mode of how AIM interacts with IgM-Fc within the 50° gap needs to be addressed three dimensionally, our findings demonstrate a unique association between AIM and the IgM pentamer. In addition to the disulfide bond between the SRCR2 domain of AIM and the Fc-Cμ3 domain at one side of the gap space, we demonstrated that the SRCR3 domain was responsible for AIM-IgM binding. Although the positively charged amino acid cluster within the SRCR3 domain likely attaches on the negatively charged area of the Cμ4 domain of the IgM-Fc, the precise mode of the charge interaction including the information of the responsible amino acids at the Cμ4 domain will need to be clarified with additional experiments. The 50° gap was unsubstitutable for AIM association, as the pentamer without J chain, which was also asymmetric but with a narrower gap than the 50° gap of J-positive pentamer, did not accept AIM. It is possible that a narrower gap may cause inappropriate positioning of the Cys^194^ at the SRCR2 domain of AIM and the Cys^414^ at the Fc-Cμ3 domain, resulting in a defective disulfide bond between the cysteine residues. One could argue that AIM might bind to IgM through a possible association with the J chain, but this is unlikely, as we previously demonstrated that AIM does not associate with the J chain (*42*). It is still unclear whether the 50° gap binds only AIM or can bind different molecules. However, because the serum levels of AIM and IgM are strongly correlated (*42*, *53*), AIM might have first priority to bind to this gap, which could occur if the space were specific for AIM binding or if AIM bound with the highest affinity, easily replacing other bound molecules.

The amended view of the pentameric IgM will be the basis of future advancements to elucidate important issues regarding IgM-mediated immune functions. In addition, it may contribute to an improved design for IgM-based antibody therapies, which have been heralded as a new wave of cancer therapies. Moreover, our discovery will shed light on the dynamic regulation of AIM function mediated by the association with or dissociation from the IgM pentamer, which will also drive the development of AIM-based disease therapies.

## MATERIALS AND METHODS

### General experimental approaches

No samples or data points were excluded from the reported analyses. Samples were not randomized to experimental groups. Analyses were not performed in a blinded fashion.

### Negative-stain EM and reference-free 2D class average analysis

For negative-stain EM, 5 μl of droplet of purified protein at approximately 15 μg/ml was applied to a glow-discharged carbon-coated grid (ELS-C10, Stem) and removed by filter paper. The grid was immediately stained using 1% uranyl acetate solution. During the staining, we placed a drop of uranyl acetate on the grid, sucked it up immediately using filter paper (without a dry up in the first and second runs), and repeated the procedure in succession. All data were acquired with a JEOL JEM-2010F electron microscope operated at 200 kV using a Tietz 4k by 4k complementary metal-oxide semiconductor camera (TemCam-F416, TVIPS). Data processing was performed using Scipion software package (*54*). Contrast transfer function (CTF) was estimated by Gctf (*55*). Xmipp3 (manual picking and auto picking) (*56*) and Gautomatch (developed by K. Zhang, MRC Laboratory of Molecular Biology, Cambridge, UK, www.mrc-lmb.cam.ac.uk/kzhang/Gautomatch/) were used for particle picking. The number of picked particles for each sample were approximately 6500 (mouse IgM-Fc), 4500 (human IgM-Fc), 3400 (native mouse IgM), 8700 (J-negative IgM-Fc), 3700 (Cys^414^Ser IgM-Fc), 14,000 (mouse AIM-IgM Fc), and 9600 (human AIM-IgM Fc). Particles were extracted with phase-flipping, and subsequently, reference-free 2D class average was performed using RELION-2.1 (*57*).

### Production and purification of IgM pentamer and recombinant AIM

Mouse and human IgM-Fc were produced as follows. HEK293T cells were cotransfected with pCAGGS-FLAG-IgM-Fc and pCAGGS–J chain–HA plasmids and then cultured in Dulbecco’s modified Eagle’s medium (DMEM), high glucose, and GlutaMAX medium (Gibco, Carlsbad, CA), supplemented with 10% fetal bovine serum (FBS) for 3 days. Pentameric FLAG-Fc protein associated with the Myc-tagged J chain was purified from culture supernatant using the ANTI-FLAG M2 Affinity Gel (Sigma-Aldrich) and then eluted with glycine-HCl (pH 2.6), followed by neutralization with 1 M tris-HCl (pH 8.5). After the affinity purification using an antibody, the protein was undergone a gel size fractionation to further eliminate undesired protein. Protein was concentrated using Amicon Ultra filter concentrators (Millipore, MA) and stored at 4°C in phosphate-buffered saline (PBS). Protein concentration was determined by a bicinchoninic acid (BCA) assay according to the manufacturer’s protocol (Pierce, Rockford, IL). The protein was then subjected to size-exclusion chromatography on the HiPrep 16/60 Sephacryl S-300 HR column (GE Healthcare Life Sciences, PA) with Dulbecco’s PBS for negative-stain EM. Mouse and human IgM-Fc (with the J chain) associated with AIM were produced as follows. HEK293T cells transfected with pCAGGS-AIM were cocultured with HEK293T cells transfected with both pCAGGS-FLAG-IgM-Fc and pCAGGS-J chain-Myc in DMEM, high glucose, and a GlutaMAX medium, supplemented with 10% FBS for 3 days. The complex was purified from culture supernatant using the ANTI-FLAG M2 Affinity Gel (Sigma-Aldrich) and then a rabbit anti-AIM polyclonal antibody (rab2, made in-house) conjugated to a HiTrap NHS-activated HP column (GE Healthcare Life Sciences, PA). The protein was eluted with 0.1 M glycine-HCl (pH 2.3) and neutralized with 1 M tris-HCl (pH 8.5). After the affinity purification using antibodies, the protein was undergone a gel size fractionation to further eliminate undesired protein such as the IgM-free AIM. Protein was concentrated using Amicon Ultra filter concentrators (Millipore, MA) and stored at 4°C in PBS. Protein concentration was determined by a BCA assay according to the manufacturer’s protocol (Pierce, Rockford, IL). Monoclonal IgM proteins were purified using the HiTrap IgM purification column (GE Healthcare Life Sciences) from the culture supernatant of B cell hybridoma (clone 7C8; HB-8465, American Type Culture Collection) according to the manufacturer’s protocol.

### Quantitative Western blotting for the AIM number associating with a pentameric IgM

HEK293T cells transfected with pCAGGS-AIM-HA were cocultured with HEK293T cells transfected with both pCAGGS-FLAG-mouse Fc and pCAGGS-mouse J chain–HA in DMEM, supplemented with 10% FBS for 1 day. From the culture supernatant, the Fc-pentamer/J-HA/AIM-HA complex was purified using the ANTI-FLAG M2 Affinity Gel (Sigma-Aldrich), followed by a rabbit anti-AIM polyclonal antibody (rab2, made in house) conjugated to a HiTrap NHS-activated HP column (GE Healthcare Life Sciences, PA). The eluate was analyzed for J chain and AIM by Western blotting in a reducing condition using an anti-HA antibody. The intensity of the signal was measured using the NIH ImageJ software.

### Homology modeling

Homology models of AIM and IgM-Fc were generated using the SWISS-MODEL server (http://swissmodel.expasy.org/). Templates with the highest quality (5a2e.1.A and 1o0v.1.B for AIM and IgM-Fc, respectively) were selected for model building. Models were built on the basis of target-template alignment and subjected to energy minimization using PRIME (Schrödinger LLC, New York, NY). Molecular graphics images were produced using the PyMOL (Schrödinger Inc., MA, USA). Colors indicate the following: Blue is positively charged, red is negatively charged, and white is neutrally charged.

## Supplementary Material

http://advances.sciencemag.org/cgi/content/full/4/10/eaau1199/DC1

## SUPPLEMENTARY MATERIALS

Supplementary material for this article is available at http://advances.sciencemag.org/cgi/content/full/4/10/eaau1199/DC1 Fig. S1. Schematic view of the conventional model for pentameric IgM. Fig. S2. Immunoblotting corresponding to Fig. 1A. Fig. S3. Nonprocessed images of the negative-stain EM. Fig. S4. Analysis profile of the negative-stain EM image for the mouse IgM-Fc with the J chain. Fig. S5. Analysis profile of the negative-stain EM image for the mouse IgM-Fc pentamer with the J chain using cisTEM and Xmipp software. Fig. S6. Analysis profile of the negative-stain EM image for the human IgM-Fc pentamer with the J chain. Fig. S7. Analysis profile of the negative-stain EM image for the mouse IgM (full length). Fig. S8. Analysis profile of the negative-stain EM image for the mouse IgM-Fc without J chain. Fig. S9. Analysis profile of the negative-stain EM image for the mouse IgM-Fc Cys^414^Ser with J chain. Fig. S10. Graphic abstract of the major findings.
