# Supplementary material for: The IgM pentamer is an asymmetric pentagon with an open groove that binds the AIM protein
Source: Sci Adv. 2018 Oct 10;4(10):eaau1199. doi: 10.1126/sciadv.aau1199 (PMC6179379; doi:10.1126/sciadv.aau1199)
Supplement: http://advances.sciencemag.org/cgi/content/full/4/10/eaau1199/DC1 [file supp_4_10_eaau1199__index.html]

Science Advances | Science Advances

## Supplementary Materials

**This PDF file includes:**

- Fig. S1. Schematic view of the conventional model for pentameric IgM.
- Fig. S2. Immunoblotting corresponding to Fig. 1A.
- Fig. S3. Nonprocessed images of the negative-stain EM.
- Fig. S4. Analysis profile of the negative-stain EM image for the mouse IgM-Fc with the J chain.
- Fig. S5. Analysis profile of the negative-stain EM image for the mouse IgM-Fc pentamer with the J chain using cisTEM and Xmipp software.
- Fig. S6. Analysis profile of the negative-stain EM image for the human IgM-Fc pentamer with the J chain.
- Fig. S7. Analysis profile of the negative-stain EM image for the mouse IgM (full length).
- Fig. S8. Analysis profile of the negative-stain EM image for the mouse IgM-Fc without J chain.
- Fig. S9. Analysis profile of the negative-stain EM image for the mouse IgM-Fc Cys414Ser with J chain.
- Fig. S10. Graphic abstract of the major findings.

Download PDF

**Files in this Data Supplement:**

- Adobe PDF - aau1199\_SM.pdf
