# Supplementary material for: The IgM pentamer is an asymmetric pentagon with an open groove that binds the AIM protein
Source: Sci Adv. 2018 Oct 10;4(10):eaau1199. doi: 10.1126/sciadv.aau1199 (PMC6179379; doi:10.1126/sciadv.aau1199)
Supplement: http://advances.sciencemag.org/cgi/content/full/4/10/eaau1199/DC1 [file aau1199_SM.pdf]

Science Advances
AAAS

advances.sciencemag.org/cgi/content/full/4/10/eaau1199/DC1

Supplementary Materials for

The IgM pentamer is an asymmetric pentagon with an open groove that binds the AIM protein

Emiri Hiramoto, Akihisa Tsutsumi, Risa Suzuki, Shigeru Matsuoka, Satoko Arai*, Masahide Kikkawa, Toru Miyazaki*

*Corresponding author. Email: tm@m.u-tokyo.ac.jp (T.M.); sarai@m.u-tokyo.ac.jp (S.A.)

Published 10 October 2018, *Sci. Adv.* **4**, eaau1199 (2018)
DOI: 10.1126/sciadv.aau1199

This PDF file includes:

Fig. S1. Schematic view of the conventional model for pentameric IgM.
Fig. S2. Immunoblotting corresponding to Fig. 1A.
Fig. S3. Nonprocessed images of the negative-stain EM.
Fig. S4. Analysis profile of the negative-stain EM image for the mouse IgM-Fc with the J chain.
Fig. S5. Analysis profile of the negative-stain EM image for the mouse IgM-Fc pentamer with the J chain using cisTEM and Xmipp software.
Fig. S6. Analysis profile of the negative-stain EM image for the human IgM-Fc pentamer with the J chain.
Fig. S7. Analysis profile of the negative-stain EM image for the mouse IgM (full length).
Fig. S8. Analysis profile of the negative-stain EM image for the mouse IgM-Fc without J chain.
Fig. S9. Analysis profile of the negative-stain EM image for the mouse IgM-Fc $Cys^{414}Ser$ with J chain.
Fig. S10. Graphic abstract of the major findings.

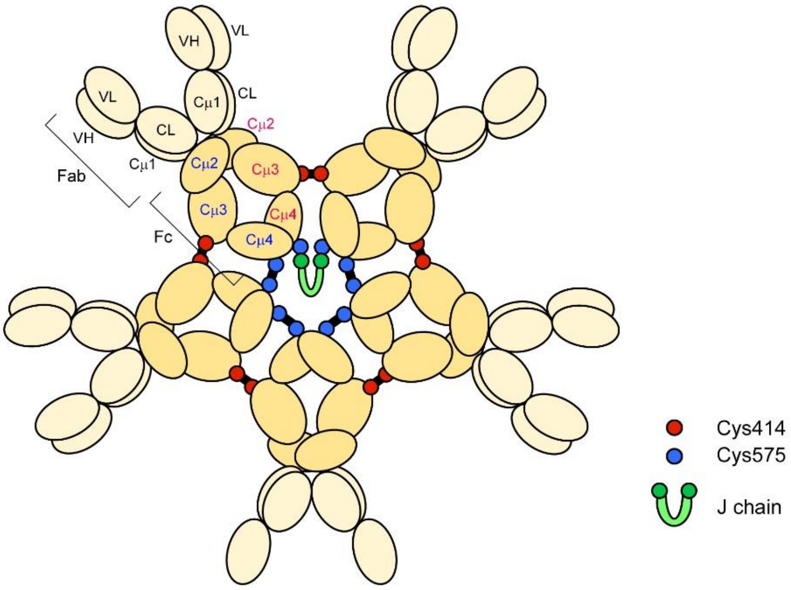

Fig. S1. Schematic view of the conventional model for pentameric IgM. The textbook conventional model for the IgM-pentamer has been a star-shaped, symmetric pentagon. Each Ig-like polypeptide (V and Cμ domain; yellow), J chain (green), and the cysteine residues involved in pentameric formation of Fc monomers (red; Cys414 at the Cμ3 domain, blue; Cys575 at the tail peptide) as well as those in the J chain (green), are presented. The disulfide bond is depicted by black line.

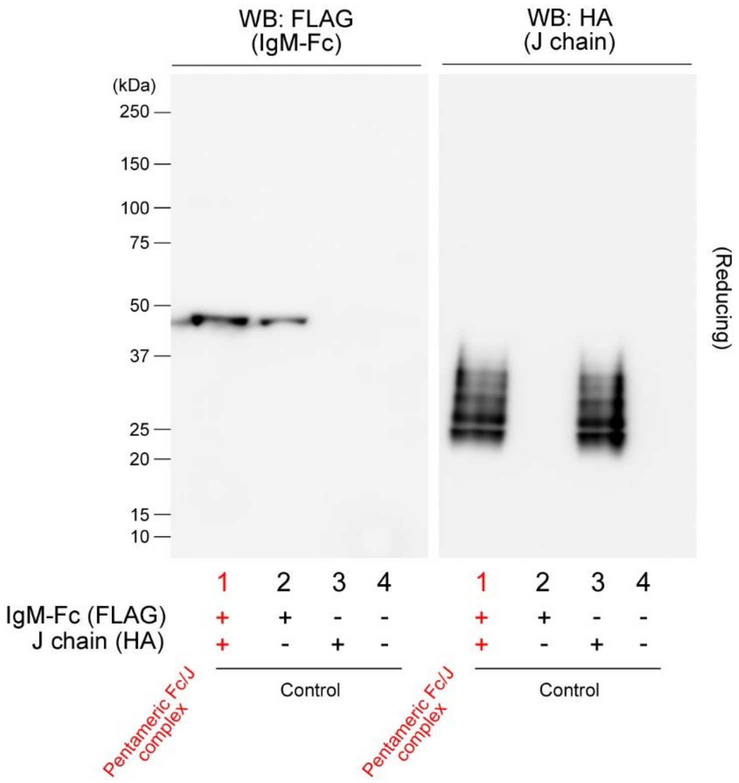

Fig. S2. Immunoblotting corresponding to Fig. 1A. The whole picture of the immunoblotting for IgM-Fc (using an anti-Flag antibody) and the J chain (using an anti-HA antibody) in a reducing condition, which is presented in Fig. 1A.

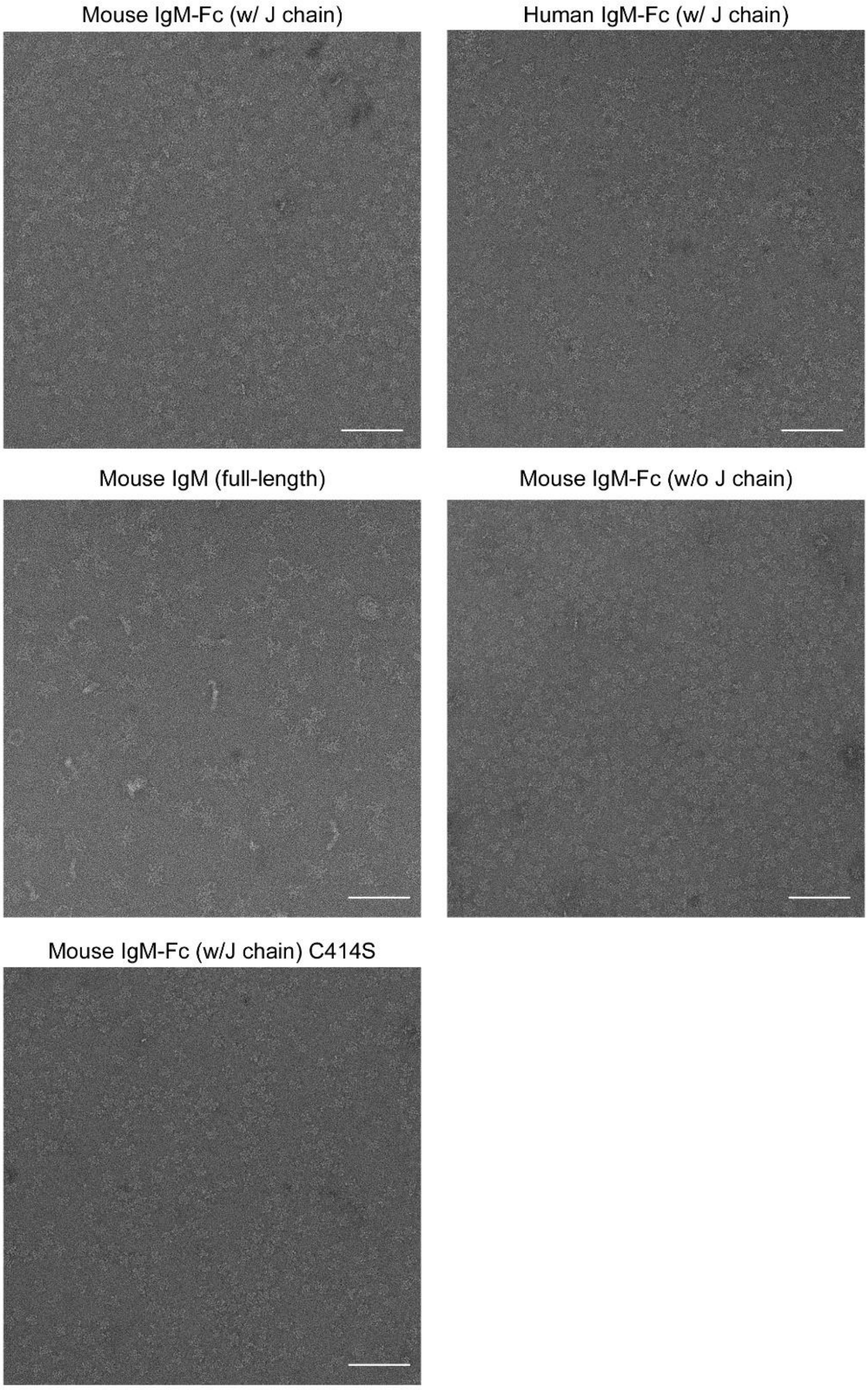
Mouse IgM-Fc (w/ J chain)
Human IgM-Fc (w/ J chain)
Mouse IgM (full-length)
Mouse IgM-Fc (w/o J chain)
Mouse IgM-Fc (w/J chain) C414S

Fig. S3. Nonprocessed images of the negative-stain EM. The non-processed images for the negative-stain EM performed in this study are presented. Scale bars: 100 nm. Mouse IgM-Fc (w/ J chain) for Fig. 1B, human IgM-Fc (w/ J chain) for Fig. 1C, mouse IgM (full-length) for Fig. 1D, mouse IgM-Fc (w/o J chain) for Fig. 2A, and mouse IgM-Fc Cys414Ser for Fig. 2B.

A **The negative-stain image for mouse IgM-Fc (w/ J chain)**

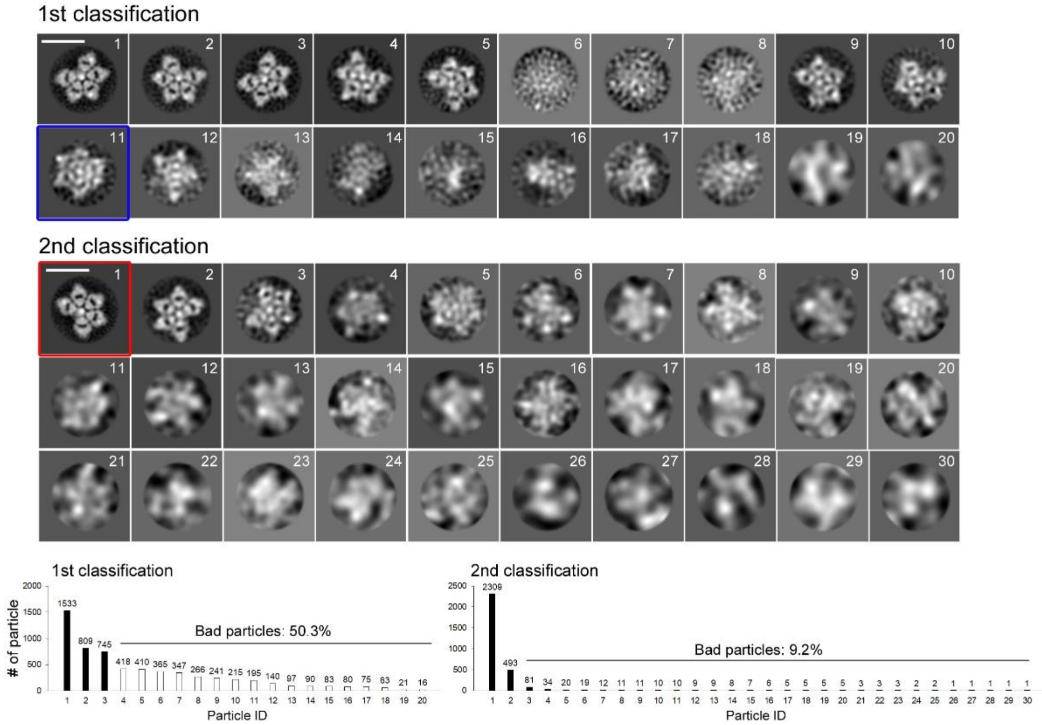

B

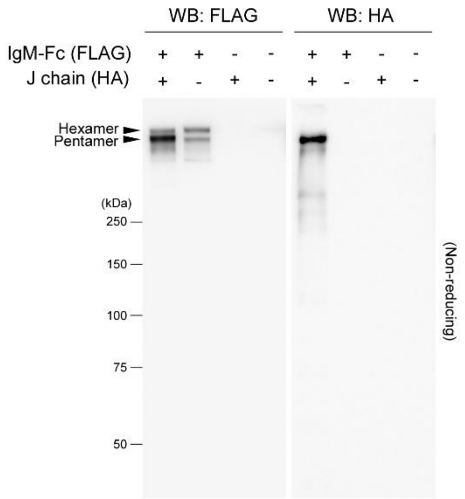

Fig. S4. Analysis profile of the negative-stain EM image for the mouse IgM-Fc with the J chain. (A) The results of the 1st classification (20 classes) and the 2nd classification (30 classes) of the images classified at higher averages in the 1st classification, are presented. The class average analysis was carried out by using Relion software. The number of individual particles represented by each class average is shown in graphs. Classes that were subjected to the 2nd classification are shown as black bars in the graph of the 1st classification, whereas classes with poor image resolution or ambiguous shape were defined as "bad particles" in the graphs of 1st and the 2nd classifications. The image presented in Fig. 1B is framed by red line. Scale bar: 20 nm. **(B)** Flag-tagged mouse IgM-Fc and HA-tagged J chain were expressed in HEK293T cells, and the supernatant was analyzed for IgM-Fc (using an anti-Flag antibody) in a non-reducing condition as in Fig. 1B. A representative blot obtained by a longer run on a gel and a short exposure at the photo shooting is presented. The IgM oligomer harbors two bands, a weak signal at the hexamer size and a strong signal at the pentamer size. This results is consistent with the observation that the hexamer-like particle was also seen at a low average in the first class analysis of the mouse IgM-Fc pentamer with the J chain (framed by blue line, fig. S4A). These results suggest that a small proportion of IgM hexmaer was formed without the J chain in the cells co-transfected with the Fc and the J expressing vectors.

The negative-stain image for mouse IgM-Fc (w/ J chain)

CisTEM

1st classification

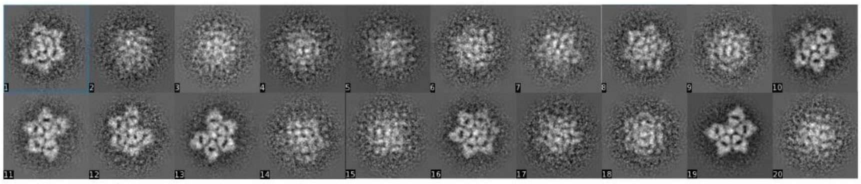

194	131	164	159	158	138	169	143	140	264
253	171	368	143	149	235	139	151	459	157

2nd classification

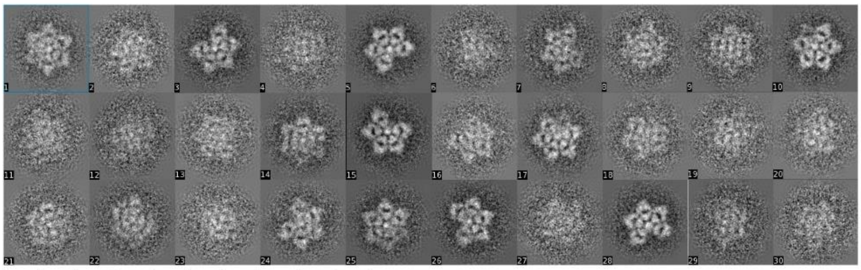

89	34	101	21	196	28	61	40	38	143
27	22	30	73	162	40	118	35	27	40
31	43	34	62	99	113	21	168	28	20

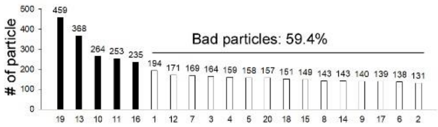

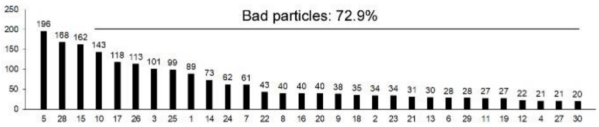

Xmipp

1st classification

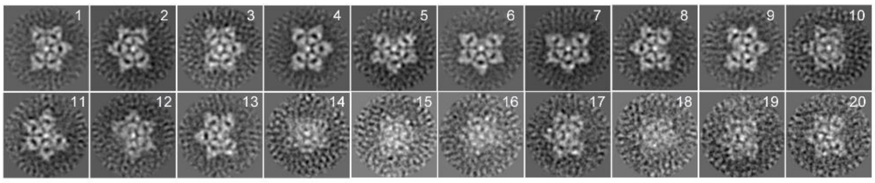

2nd classification

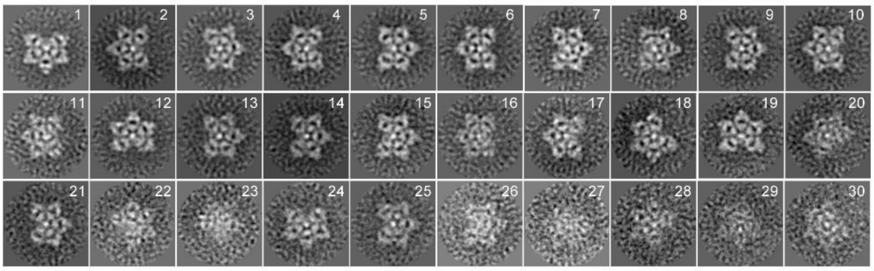

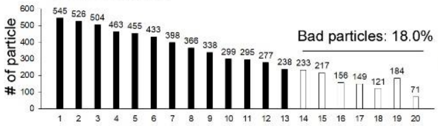

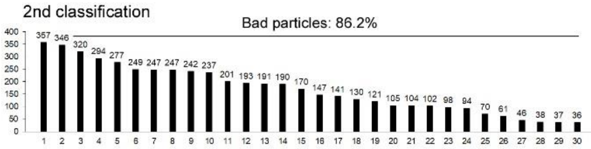

Fig. S5. Analysis profile of the negative-stain EM image for the mouse IgM-Fc pentamer with the J chain using cisTEM and Xmipp software. The individual particles of the mouse IgM-Fc with the J chain were re-analyzed by using cisTEM (upper panels) and Xmipp (lower panels) software. Similar results were obtained as that in Relion analysis (Fig. 1B, fig. S4A). In the gallery obtained by cisTEM, the classification is not arranged in descending order, and the number of individual particles are presented underneath. The number of individual particles represented by each class average is shown in graphs. Classes that were subjected to the 2nd classification are shown as black bars in the graph of the 1st classification, whereas classes with poor image resolution or ambiguous shape were defined as “bad particles” in the graphs of 1st and the 2nd classifications.

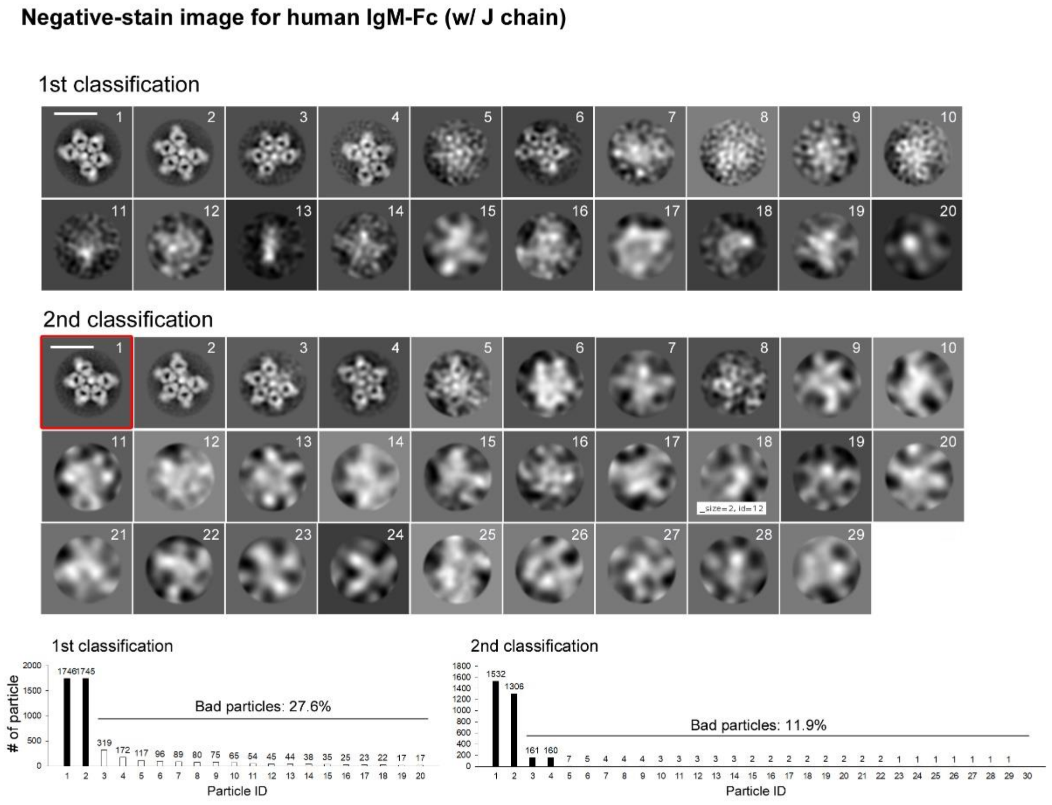

Fig. S6. Analysis profile of the negative-stain EM image for the human IgM-Fc pentamer with the J chain. The results of the 1st classification (20 classes) and the 2nd classification (30 classes) of the images classified at higher averages in the 1st classification, are presented. The class average analysis was carried out by using Relion software. The number of individual particles represented by each class average is shown in graphs. Classes that were subjected to the 2nd classification are shown as black bars in the graph of the 1st classification, whereas classes with poor image resolution or ambiguous shape were defined as “bad particles” in the graphs of 1st and the 2nd classifications. The representative image presented in Fig. 1C is framed by red line. Scale bar: 20 nm.

A The negative-stain image for mouse IgM (full-length) analyzed by Relion

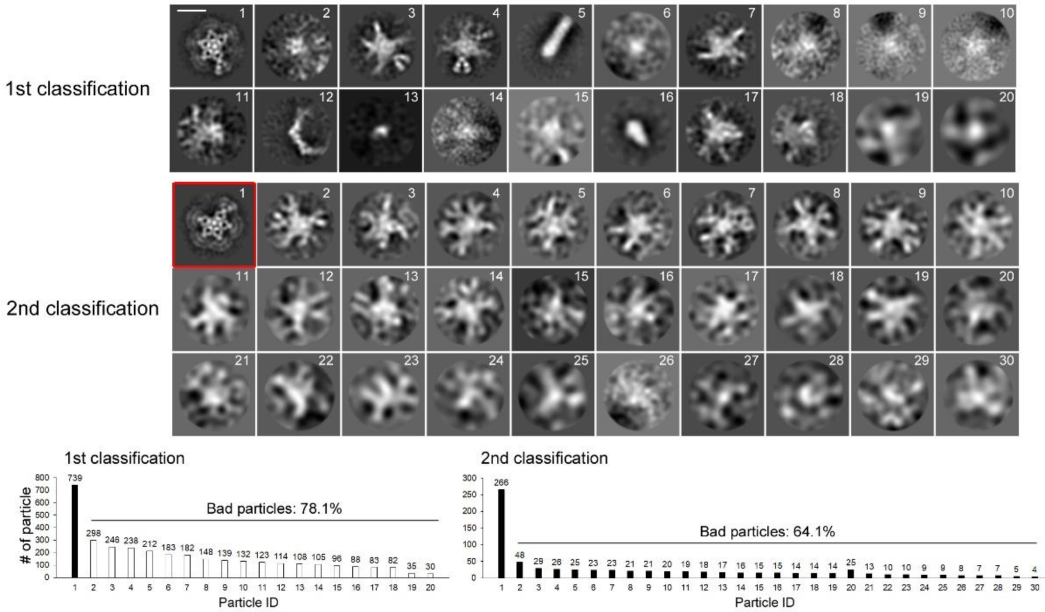

B The negative-stain image for mouse IgM (full-length) analyzed by CisTEM

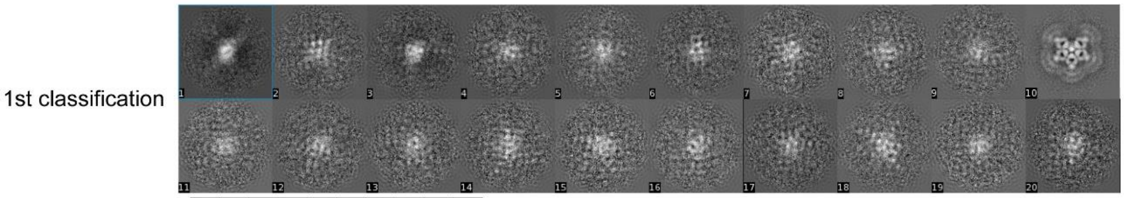

185	153	141	134	112	149	130	123	158	730
132	119	131	121	114	122	135	124	228	140

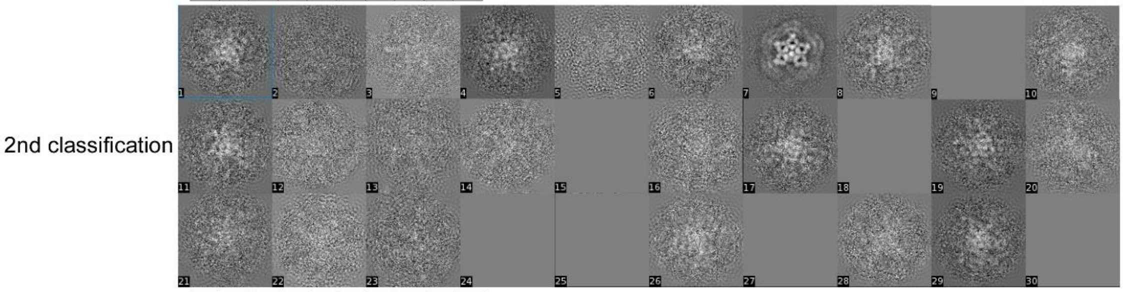

46	3	5	52	2	14	291	40	0	26
34	5	5	4	0	9	43	0	39	12
13	6	3	0	0	24	0	10	44	0

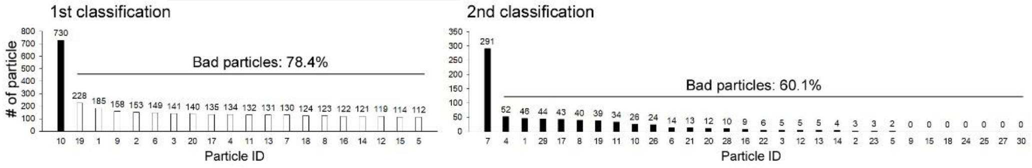

Fig. S7. Analysis profile of the negative-stain EM image for the mouse IgM (full length). (A) The results of the 1st classification (20 classes) and the 2nd classification (30 classes) of the images classified at higher averages in the 1st classification, are presented. The class average analysis was carried out by using Relion. The number of individual particles represented by each class average is shown in graphs. Classes that were subjected to the 2nd classification are shown as black bars in the graph of the 1st classification, whereas classes with poor image resolution or ambiguous shape were defined as “bad particles” in the graphs of 1st and the 2nd classifications. The representative image presented in Fig. 1D is framed by red line. Scale bar: 20 nm. **(B)** The individual particles of the mouse full length IgM-Fc (with the J chain) were re-analyzed by using cisTEM software. A similar result was obtained as that in Relion analysis (Fig. 1D, fig. S7A).

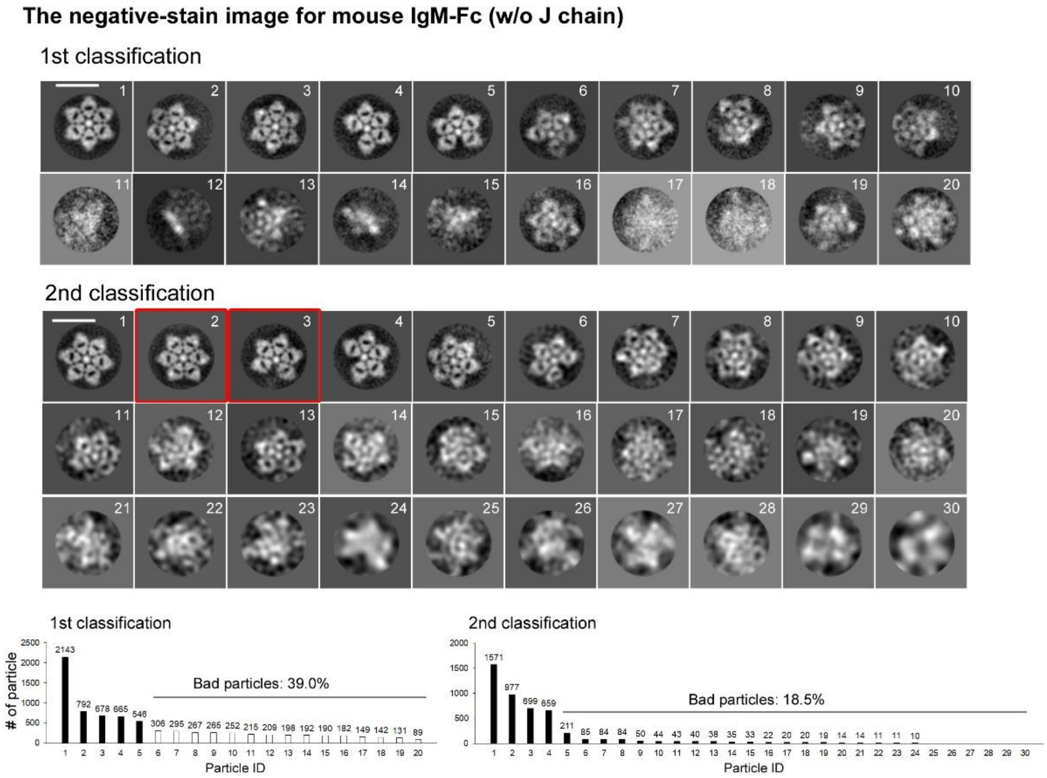

Fig. S8. Analysis profile of the negative-stain EM image for the mouse IgM-Fc without J chain. The results of the 1st classification (20 classes) and the 2nd classification (30 classes) of the images classified at higher averages in the 1st classification, are presented. The class average analysis was carried out by using Relion. The number of individual particles represented by each class average is shown in graphs. Classes that were subjected to the 2nd classification are shown as black bars in the graph of the 1st classification, whereas classes with poor image resolution or ambiguous shape were defined as “bad particles” in the graphs of 1st and the 2nd classifications. The representative images presented in Fig. 2A are framed by red line. Scale bar: 20 nm.

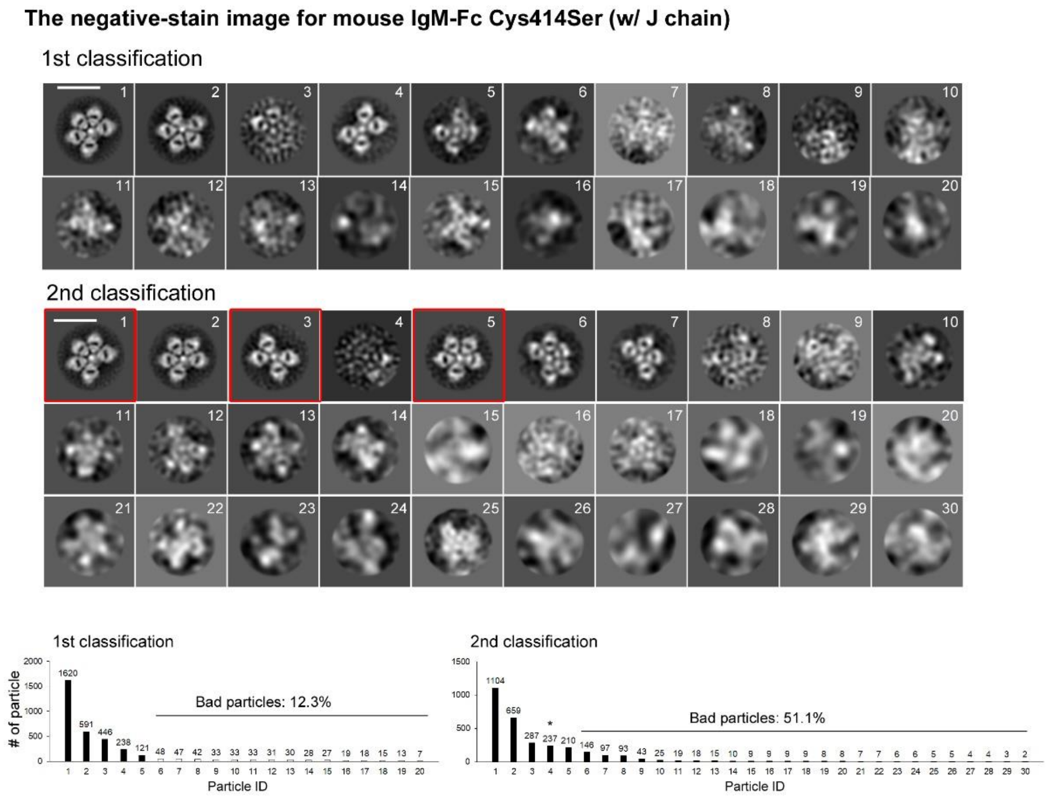

Fig. S9. Analysis profile of the negative-stain EM image for the mouse IgM-Fc Cys^{414}Ser with J chain. The results of the 1st classification (20 classes) and the 2nd classification (30 classes) of the images classified at higher averages in the 1st classification, are presented. The class average analysis was carried out by using Relion. The number of individual particles represented by each class average is shown in graphs. Classes that were subjected to the 2nd classification are shown as black bars in the graph of the 1st classification, whereas classes with poor image resolution or ambiguous shape were defined as “bad particles” in the graphs of 1st and the 2nd classifications. Note that the particle #4 is also a bad particle, which is indicated by asterisk. The representative images presented in Fig. 2B are framed by red line. Scale bar: 20 nm.

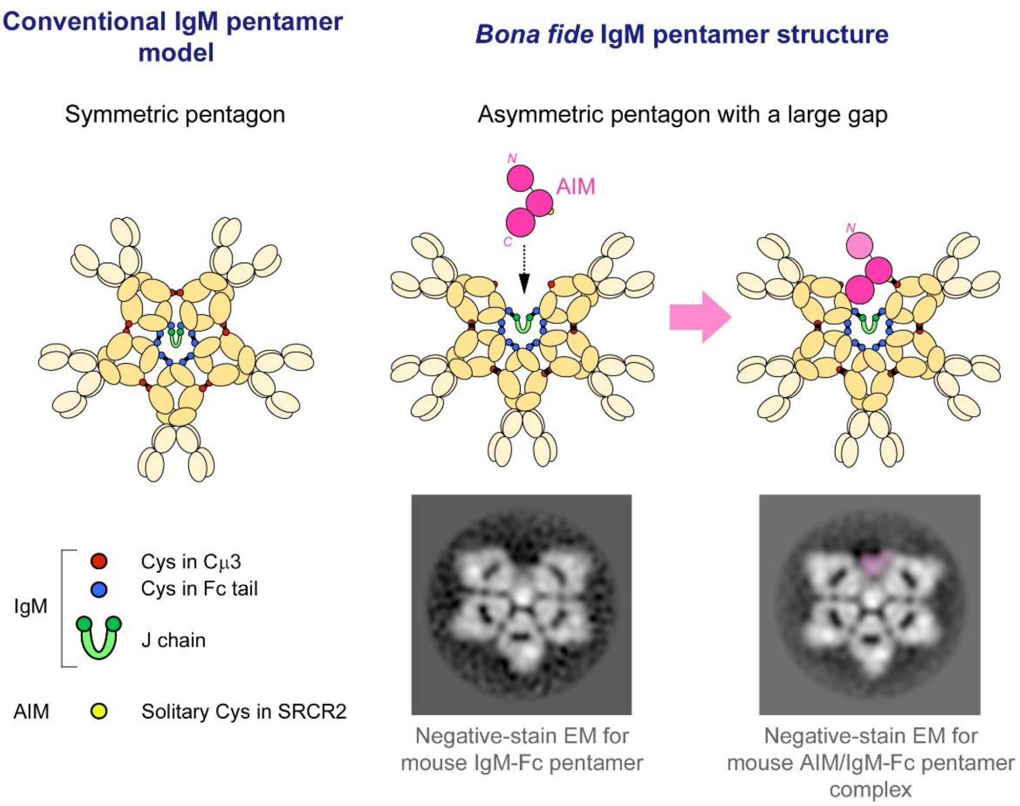

Fig. S10. Graphic abstract of the major findings. The textbook conventional model of the IgM pentamer has been a star-shaped, symmetric pentagon (left). However, our current study revises the model to an asymmetric pentagon with a large structural gap (middle). A single AIM molecule fits into the gap, cross-bridging the two IgM-Fc consisting the edges of the gap through a disulfide bond and a charge-based interaction (right).
